# Performance of computerized self-reported medical history taking and HEAR score for safe early rule-out of cardiac events in acute chest pain patients: the CLEOS-CPDS prospective cohort study

**DOI:** 10.1093/ehjdh/ztae087

**Published:** 2024-11-12

**Authors:** Helge Brandberg, Fanny Schierenbeck, Carl Johan Sundberg, Sabine Koch, Jonas Spaak, Thomas Kahan

**Affiliations:** Division of Cardiovascular Medicine, Department of Clinical Sciences, Danderyd Hospital, Karolinska Institutet, SE-182 88 Stockholm, Sweden; Division of Cardiovascular Medicine, Department of Clinical Sciences, Danderyd Hospital, Karolinska Institutet, SE-182 88 Stockholm, Sweden; Department of Learning, Informatics, Management and Ethics, Karolinska Institutet, SE-171 77 Stockholm, Sweden; Department of Physiology and Pharmacology, Karolinska Institutet, SE-171 77 Stockholm, Sweden; Department of Learning, Informatics, Management and Ethics, Karolinska Institutet, SE-171 77 Stockholm, Sweden; Division of Cardiovascular Medicine, Department of Clinical Sciences, Danderyd Hospital, Karolinska Institutet, SE-182 88 Stockholm, Sweden; Division of Cardiovascular Medicine, Department of Clinical Sciences, Danderyd Hospital, Karolinska Institutet, SE-182 88 Stockholm, Sweden

**Keywords:** Chest pain, Risk assessment, Coronary artery disease, Medical informatics, Artificial intelligence, Medical history taking

## Abstract

**Aims:**

A simplified version of the history, electrocardiogram, age, risk factors, troponin (HEART) score, excluding troponin, has been proposed to rule-out major adverse cardiac events (MACEs). Computerized history taking (CHT) provides a systematic and automated method to obtain information necessary to calculate the HEAR score. We aimed to evaluate the efficacy and diagnostic accuracy of CHT in calculating the HEAR score for predicting MACE.

**Methods and results:**

Prospective study including clinically stable adults presenting with chest pain at the emergency department (ED) of Danderyd University Hospital (Stockholm, Sweden), in 2017–19. Participants entered their medical histories on touchscreen tablets using CHT software. The HEAR and HEART scores were calculated from CHT data. Thirty-day MACE and acute coronary syndrome (ACS) outcomes were retrieved, and the diagnostic accuracy was assessed. Logistic regression was used to determine the most predictive components of the HEAR score. Among 1000 patients, HEART and HEAR scores could be calculated from CHT data in 648 and 666 cases, respectively, with negative predictive values [95% confidence interval (CI)] of 0.98 (0.97–0.99) and 0.99 (0.96–1.00). Two patients with HEAR score <2 experienced a 30-day MACE. The age [odds ratio (OR) 2.75, 95% CI 1.62–4.66] and history (OR 2.38, 95% CI 1.52–3.71) components of the HEAR score were most predictive of MACE. Acute coronary syndrome outcomes provided similar results.

**Conclusion:**

The HEAR score acquired by CHT identifies very-low-risk patients with chest pain in the ED, safely ruling out ACS and MACE. This highlights the value of computerized history taking by patients, which may reduce unnecessary tests and hospital admissions.

**Trial Registration:**

ClinicalTrials.gov NCT03439449.

## Graphical Abstract

**Graphical Abstract:**
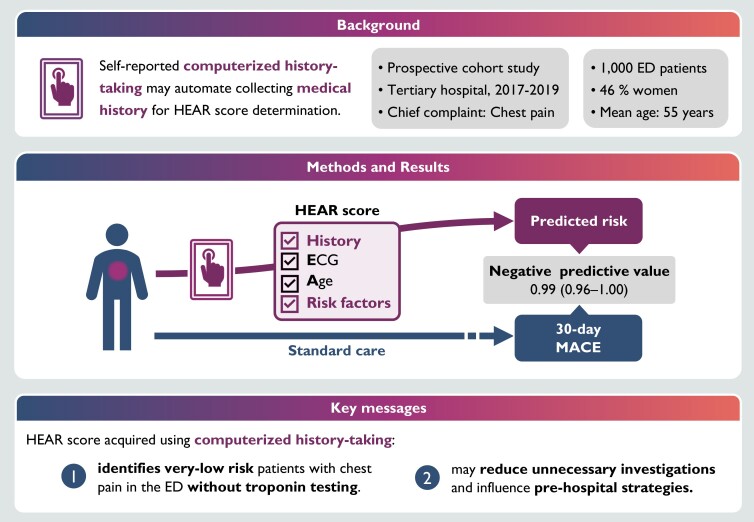

## Introduction

Chest pain is a common symptom in emergency departments (EDs), with causes ranging from emergent to less acute conditions. Globally, about 1 in 10 ED chest pain patients are diagnosed with acute coronary syndrome (ACS).^1,2^ To assist in clinical decision-making for chest pain patients, international guidelines recommend risk stratification scores that integrate medical history, clinical signs and symptoms, electrocardiogram (ECG) data, and cardiac troponins to predict the risk of a major adverse cardiac event (MACE), encompassing (i) a diagnosis of ACS, (ii) revascularization, or (iii) cardiovascular death within 30 days.^3,4^ The history, ECG, age, risk factors, and troponin (HEART) score^5,6^ has substantial scientific support^7^ and has proven effective in increasing the proportion of patients eligible for early discharge.^8,9^ However, the use of chest pain risk scores in the ED is inconsistent,^10^ and subject to variation in risk calculations among clinicians.^11,12^ Furthermore, concerns have been raised regarding the potential overuse of troponin testing, contributing to unnecessary delays in already overburdened EDs.^13^ To facilitate rapid discharge of low-risk chest pain patients, without relying on troponin testing, a simplified HEART score, known as the HEAR score, has been evaluated across multiple studies.^14–16^ Such approach could identify very-low-risk patients who could be safely sent home after triage, while also identifying patients appropriate for further work-up, including troponin testing, for those at higher risk. The HEAR score has also been validated in pre-hospital settings, suggesting a broader applicability in emergency medical services.^17^

Computerized history taking (CHT), a method for automating the collection of medical histories, could aid the clinician in systematically gathering the required data and integrate risk stratification scores, such as the HEAR score, to improve clinical decision-making. Computerized history taking has been studied in acute care settings,^18,19^ demonstrating feasibility for determining HEART score^20,21^ and high patient acceptance.^22^ We hypothesize that using self-reported CHT data to calculate the HEAR score is an effective method for risk stratification in the ED. This could facilitate a more rapid and resource-efficient triage, safely rule-out MACE, and prioritize care for patients at higher risk.

The objectives of this study were to (i) determine the efficacy of CHT to calculate the HEAR score in patients presenting to the ED due to acute chest pain, (ii) evaluate the diagnostic accuracy of this score to safely rule-out 30-day MACE or ACS, and (iii) identify the key components in the HEAR score that are most predictive of such outcomes.

## Methods

### Study design and setting

The Clinical Expert Operating System Chest Pain Danderyd Study (CLEOS-CPDS; ClinicalTrials.gov identifier: NCT03439449) is a prospective cohort study, aiming to determine the value of self-reported CHT in the management of acute chest pain.^23^ Patients presenting to the ED at Danderyd University Hospital, a tertiary hospital in Stockholm, Sweden, were recruited. In this study, we present an analysis of the first 1000 patients enrolled as pre-specified in the CLEOS-CPDS study protocol,^23^ from 1 October 2017 to 16 May 2019. This exploratory study calculated the sample size based on the desired precision for sensitivity and specificity, as previously outlined.^23^ Since the prevalence of ACS in the study population was unknown, we assumed a prevalence of 50% to maximize the estimated sample size. To achieve a precision of ±0.03 (3%) for both sensitivity and specificity (using nQuery V.7.0; Statistical Solutions, Boston, MA, USA), a total of 1000 patients was required. As sensitivity or specificity approaches the extremes (closer to 0 or 1), the required sample size decreases due to increased precision.

This study complies with the Standards for Reporting Diagnostic Accuracy (STARD 2015) guidelines,^24^ adheres to the Declaration of Helsinki and was approved by the Stockholm Regional Ethical Committee (currently known as Swedish Ethical Review Authority), reference number 2015/1955-1. Informed consent was obtained from all participants prior to their inclusion.

Details on patient disposition and eligibility criteria have been described.^21,23^ Briefly, patients presenting in the ED with acute chest pain were triaged by cardiology consultants or senior residents from 08:00 to 17:00, and by triage nurses using the Rapid Emergency Triage and Treatment System (RETTS) protocol^25^ from 17:00 to 08:00. Based on the triage outcome, patients were directed either to the 24 h ED cardiology unit, staffed by consultants in cardiology or senior residents, or to the cardiology inpatient day-care unit, operational daily from 08:00 to 17:00. The latter functions as an observational day ward and is staffed by cardiology consultants who have easy access to both invasive and non-invasive cardiac imaging. Before admission to either the ED cardiology unit or the inpatient day-care unit, ECG, and biomarkers were collected.

Patients eligible for inclusion were adults (≥18 years) with a chief complaint of chest pain, adequate knowledge in Swedish, clinical stability (RETTS level orange, yellow, green, and blue), and a first ECG not indicative for ACS,^3^ e.g. absence of ST-segment elevation or ST-segment elevation equivalents. Patients were excluded if they were unable to undergo CHT, e.g. agitation, severe visual impairment, or confusion. In the ED cardiology unit or cardiology inpatient day-care unit, eligible patients were invited by a research assistant to participate in the study, as previously detailed.^21,23^

Upon consenting, participants were given a tablet equipped with the CHT software. A research assistant ensured that patients could manage the initial demographic questions of the CHT interview independently; those unable were excluded from the study. The CHT was conducted during waiting periods, which could occur before or after physician consultations, and could be paused for clinical procedures, e.g. blood sampling or radiographic examinations and resumed afterwards. The CHT could end at the patient’s discretion, upon ward admission, or discharge from the ED. The data from the CHT, as well as the calculated risk scores, were not available to the treating physician or other healthcare professionals and were used exclusively for clinical research purposes. All reasons for CHT discontinuation were documented by the research staff, and the CHT did not interfere with standard patient management. Patients were informed that participation in the study would neither affect waiting times nor usual care in the ED.

### Interventions

#### Computerized history taking

**Figure 1:**
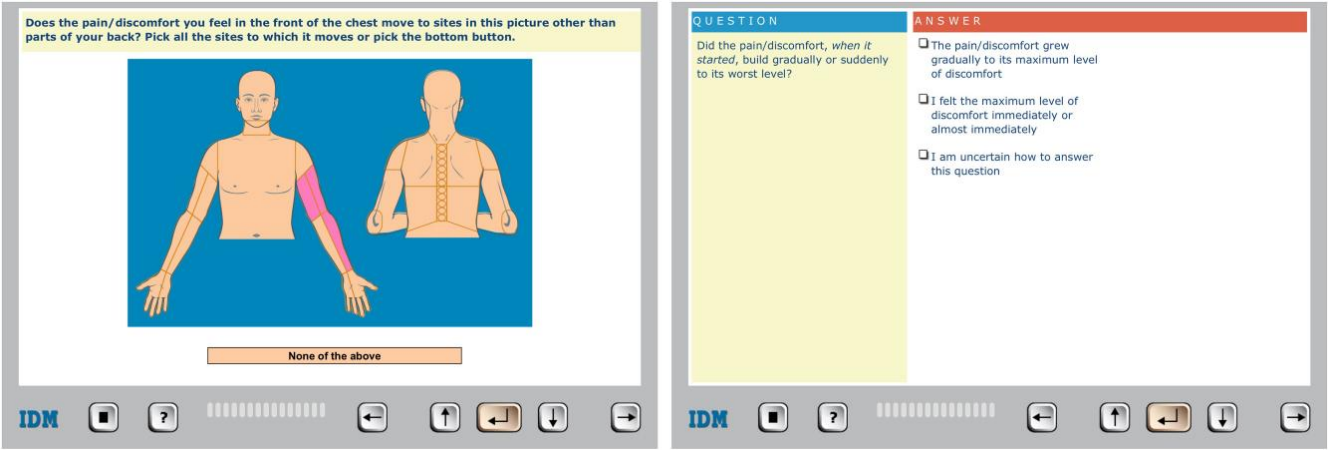
User interface and example questions in the computerized history-taking programme.

Medical histories were collected using the CHT software CLEOS, running on tablets (iPad; Apple Inc, Cupertino, CA, USA), a system previously described in detail.^23,26^ In short, CLEOS is an artificial intelligence expert system^27^ based on software algorithms representing medical knowledge as branched chain decision trees to acquire histories, including demographics, present illness, organ system, medications, and socio-economic factors. Each tree is further broken down, e.g. the cardiovascular decision tree includes separate trees for hypertension, heart failure, and arrhythmias. The software includes 29 trees specifically for chest pain assessment. Using a rule-based approach with >17 000 decision nodes, patients are guided through structured questions, predominantly in text format (e.g. yes/no, multiple choice), along with image-based questions for anatomical data, such as the locations or radiation of chest pain (*Figure 1*). Gating mechanisms prevent redundant questions by directing the interview based on prior responses. This allows the software to dynamically adapt to each patient’s responses, tailoring the interview by selecting clinically relevant questions, emulating physician-like clinical reasoning. In a validation study, we found high agreement between CHT-collected and physician-acquired HEART score variables for traditional risk factors (e.g. hypertension or diabetes mellitus), but low to moderate for chest pain characteristics (e.g. relief by rest or diaphoresis).^28^ However, CHT provided more complete data for risk stratification, suggesting its potential in the acute setting. Previous studies have shown that CLEOS provides more comprehensive medical histories for non-emergent hospitalized patients^29^ and improves adherence to dyslipidaemia guidelines.^30^

The CHT interview starts with questions concerning the chief complaint and then systematically guides the patient through a medical interview, covering demographics, present illness, organ systems, medications, and socio-economic factors. For exploratory purposes, the interview is extensive, and it may not be feasible for some patients to complete during their waiting time in the ED. Given the study’s focus on chest pain management, the initial segment of the CHT interview was specifically designed to include questions about chest pain characteristics and established risk factors for ACS. As previously reported, the median time required to collect the HEART score was 23 min (interquartile range 18–31 min).^21^

#### HEAR score

The HEAR score is based on chest pain characteristics, age, ECG findings, and risk factors. Adding troponin yields the HEART score (*Table 1*). In this study, CHT was used to retrieve data for calculating the history, age, and risk factor components of the HEAR score. The history component was scored according to regional guidelines,^31^ using the traditional clinical classification of suspected angina symptoms, i.e. (i) central chest pain, (ii) triggered by physical or emotional exertion, and (iii) relieved by rest or nitrates. Based on the number of criteria met, patient history was categorized as highly (three criteria met), moderately (two criteria met), or slightly suspicious (none or one criterion met). To compare the HEAR score with the more validated HEART score,^32^ troponin values collected according to the European Society of Cardiology 0/1 h algorithm^33^ were obtained from the electronic health record (EHR). Consequently, the index tests were the HEART and HEAR scores, calculated using CHT data.

**Table 1:** Details of the HEART and HEAR scores

Variable	Grading	Score
History	Highly suspicious	2
	Moderately suspicious	1
	Slightly or non-suspicious	0
ECG	Significant ST-depression	2
	Non-specific	1
	Normal	0
Age, years	≥65	2
	>45–64	1
	≤45	0
Risk factors	≥3 risk factors	2
	1 or 2 risk factors	1
	No risk factors	0
Troponin	≥3× normal limit	2
	1–3× normal limit	1
	Normal	0

### Outcomes

The primary outcome, which was also considered as the reference test, was defined as a 30-day MACE. To facilitate comparisons with other chest pain studies, we also report ACS as a separate outcome. According to the study protocol, an ACS diagnosis was verified by a cardiologist using European guidelines.^34,35^ The cardiologist then assigned the appropriate International Statistical Classification of Diseases and Related Health Problems, 10th Revision (ICD-10), codes based on type of ACS. In this study, ASC diagnosis was identified using ICD-10 codes I20.0 (unstable angina pectoris), I21.0-9 (acute myocardial infarction), and I24.0-9 (other acute ischaemic heart diseases).^36,37^

### Data collection

Data relevant to this study were extracted from the EHR (TakeCare, CompuGroup Medical Sweden AB, Solna, Sweden) and from the CHT database by research staff. Demographic details, including age and sex, of the general ED population at Danderyd University Hospital during the study period were retrieved from the EHR system, using QlikView version 12.10 (QlikTech International AB, Lund, Sweden).

Physician-interpreted ECG findings were obtained from the EHR. If missing (*n* = 58), automated ECG interpretations (EC Sense ECG; Cardiolex Medical AB, Stockholm, Sweden) were used. Blood samples were analysed for plasma high-sensitivity cardiac troponin T levels using the Elecsys assay (Roche Diagnostics, Basel, Switzerland) at the Karolinska University Laboratory, Stockholm. The assay has a detection limit of 5 ng/L and a 99th percentile value of 14 ng/L in healthy individuals.^38^

### Statistical analysis

Descriptive statistics are reported as mean values  ± SD or 95% confidence intervals (CIs), or proportions, as appropriate. Group differences for a 30-day MACE or ACS were assessed using Student’s *t*-test for continuous variables, and Pearson χ^2^ test for binomial and categorical variables. A two-stage logistic regression model was used to determine the most predictive components of the HEAR score for 30-day MACE. Univariate analyses were performed for each variable to identify its individual association with the reference test. Subsequently, the predefined HEAR score variables were included in a multivariable model to account for potential confounding factors and assess their relative contributions to the overall predictive ability of the score. Spearman’s rank correlation coefficient was used to evaluate the association between HEAR score variables and 30-day MACE. Diagnostic accuracy was evaluated by cross-tabulating index test results against the reference test, calculating sensitivity, specificity, positive predictive value, and negative predictive value (NPV). Pearson χ^2^ test was used to compare NPVs across index tests. Receiver operating characteristic curves were created for each index test, and the Hanley and McNeil method^39^ was used to test for differences. A dropout analysis was performed to identify differences between groups with complete and incomplete HEAR scores. Patients lacking data to calculate a complete score were excluded from further analysis. Analyses were performed using STATA software, version 14.2 (StataCorp, College Station, TX, USA).

## Results

### Characteristics of study subjects

**Figure 2:**
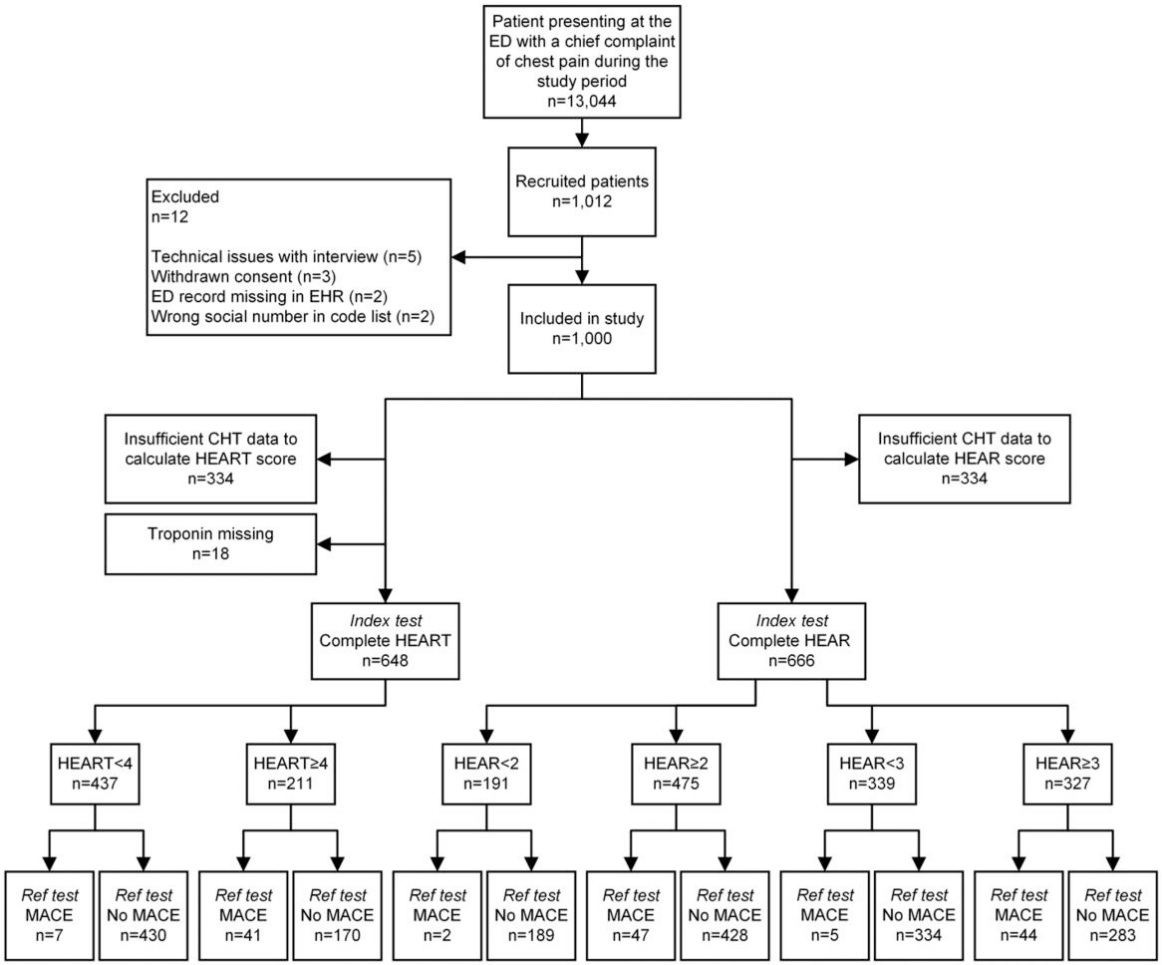
Study flow diagram. ED, emergency department; ECG, electrocardiogram; CHT, computerized history taking; Ref test, reference test. During the study period, 13 044 patients presented at the emergency department with chest pain, of which 1012 provided informed consent and met the inclusion criteria when research staff were on duty. Twelve patients were excluded, and 1000 patients were ultimately included in the study. Insufficient computerized history-taking data indicate that the patient did not complete the computerized history taking interview far enough to provide the necessary data for HEAR or HEART score calculations (see Supplementary material online, *Figure S1* for details).

During the study period, 13 044 patients with chest pain presented at the ED of Danderyd University Hospital. Of these, 1000 unselected patients were prospectively included when research staff were on duty (office hours, evenings, and weekends; not night-time). Reasons for non-inclusion are detailed in a previous study.^21^ Complete data were available to calculate the HEART score for 648 patients and the HEAR score for 666 patients (*Figure 2*). Supplementary material online, *Figure S1* illustrates missing data hindering HEAR and HEART score calculations, primarily due to incomplete CHT interviews, where 312 patients (31%) did not progress far enough to provide necessary information for the history and/or risk score components. Main reasons for discontinuing the CHT interview were that the patient felt tired (26%) or was discharged from the ED, as previously reported in detail.^28^

Baseline demographic and clinical characteristics are presented in *Tables 2* and *3* for MACE and in Supplementary material online, *Tables S1* and *S2* for ACS. Age and gender distribution (mean age 54.7 ± 17.2 years; 46% female) were similar to the general chest pain population (mean age 57.6 ± 19.1 years; 49% female) at the Danderyd University Hospital ED during the study period. Serial troponin testing was performed in 483 (48%) patients. Additionally, 528 (53%) patients were admitted to either the day-care unit or a ward due to suspected ACS or other conditions requiring further observation and evaluation. In the study population, 72 (7%) patients had a 30-day MACE, including ACS (*n* = 65) and revascularization without ACS (*n* = 7). No cases of cardiovascular death within 30 days were reported. The patients with MACE or ACS were generally older, more frequently treated for hyperlipidaemia and hypertension, had a history of coronary artery disease, new ECG changes, and higher troponin levels. Notably, ongoing chest pain during the CHT interview was more common in patients without MACE or ACS. These patients were younger (mean age 50 ± 16 vs. 59 ± 16 years; *P* < 0.01) with no differences between genders (*P* = 0.27). Current smoking was not associated with a 30-day MACE. Current smokers were younger (mean age 48 ± 16 vs. 54 ± 16 years; *P* < 0.01) and more often male (14 vs. 9%; *P* = 0.03), than non-smokers. Patients arriving at ED by ambulance were older (mean age 59 ± 17 vs. 53 ± 16 years, *P* < 0.01), with no differences between genders (*P* = 0.40).

**Table 2:** Baseline demographic of the study population, divided into groups depending on major adverse cardiac event or not

	All		MACE		Non-MACE		*P*-value
Characteristic	Value	*n*	Value	*n*	Value	*n*	
Age, years	54.7 ± 17.2	1000	67.6 ± 11.0	72	53.7 ± 17.1	928	<0.001
Sex (females)	456 (46)	1000	19 (26)	72	437 (47)	928	0.001
Body mass index, kg/m^2^	26.4 ± 4.7	1000	26.6 ± 4.3	72	26.3 ± 4.7	928	0.627
Diabetes mellitus Type 1 or 2	62 (8)	788	8 (16)	51	54 (7)	737	0.032
Ongoing lipid-lowering medication	128 (20)	640	21 (48)	44	107 (18)	596	<0.001
Hypertension	316 (41)	765	34 (64)	53	282 (40)	712	<0.001
Family history of coronary artery disease	200 (26)	774	18 (38)	48	182 (25)	726	0.057
Known coronary artery disease	137 (16)	851	31 (55)	56	106 (13)	795	<0.001
History of angina pectoris	88 (10)	850	23 (41)	56	65 (8)	794	<0.001
History of myocardial infarction	82 (10)	850	21 (38)	56	61 (8)	794	<0.001
History of percutaneous coronary intervention	76 (9)	833	16 (29)	55	60 (8)	778	<0.001
History of coronary artery bypass graft	18 (2)	833	10 (18)	55	8 (1)	778	<0.001
Current smoker	90 (11)	790	5 (9)	53	85 (12)	737	0.642
Region of birth							
Nordic countries	829 (83)	1000	63 (8)	72	766 (83)	928	0.282
Europe (outside the Nordic countries)	46 (5)	1000	1 (1)	72	45 (5)	928	0.177
Outside Europe	125 (13)	1000	8 (11)	72	117 (13)	928	0.711
Occupational status							
Active worker (employed, student)	616 (62)	1000	26 (36)	72	590 (64)	928	<0.001
Not at work (unemployed, on sick leave)	69 (7)	1000	1 (1)	72	68 (7)	928	0.055
Retired	315 (32)	1000	45 (63)	72	270 (29)	928	<0.001
Arrived at ED by ambulance	189 (21)	917	14 (24)	58	175 (20)	859	0.493
Ongoing chest pain during CHT	544 (61)	892	19 (35)	55	525 (63)	837	<0.001

Data are presented as mean values ± SD or *n* (%), as appropriate. Self-reported medical history data derived from CHT. MACE, 30-day major adverse cardiac event; CHT, computerized history taking; ED, emergency department.

**Table 3:** Vital signs, electrocardiogram, circulating biomarkers, and disposition of the study population, divided into groups depending on major adverse cardiac event or not

	All		MACE		Non-MACE		*P*-value
Characteristic	Value	*n*	Value	*n*	Value	*n*	
Vital parameters at triage							
Systolic blood pressure, mmHg	143 ± 22	995	148 ± 22	72	143 ± 22	923	0.056
Diastolic blood pressure, mmHg	83 ± 13	993	81 ± 14	72	84 ± 13	921	0.178
Heart rate	77 ± 16	984	74 ± 15	72	77 ± 16	912	0.151
Respiration rate	16 ± 3	984	16 ± 2	72	16 ± 3	912	0.849
Body temperature (°C)	36.8 ± 0.4	947	36.6 ± 0.4	70	36.8 ± 0.4	877	<0.001
ECG							
New signs diagnostic for ischaemia	68 (7)	994	14 (20)	71	54 (6)	923	<0.001
New non-specific ST-T changes	131 (13)	994	20 (28)	71	111 (12)	923	<0.001
Normal or known ST-T alterations	795 (80)	994	37 (52)	71	758 (82)	923	<0.001
High-sensitive troponin T values							
>3 × normal limit (>42 ng/L)	47 (5)	970	29 (41)	71	18 (2)	899	<0.001
1–3 × normal limit (15–42 ng/L)	112 (12)	970	19 (27)	71	93 (10)	899	<0.001
Normal limit^a^ (≤14 ng/L)	811 (84)	970	23 (32)	71	788 (88)	899	<0.001
5–14 ng/L	397 (41)	970	15 (21)	71	367 (41)	899	0.001
1 h troponin T elevated (>2 ng/L)	21 (2)	970	2 (3)	71	19 (2)	899	0.695
<5 ng/L	414 (43)	970	4 (6)	71	410 (46)	899	<0.001
Admitted to the ward or day-care unit	528 (53)	990	69 (96)	72	459 (50)	918	<0.001
Ward (not via day-care unit)	203 (21)	990	61 (85)	72	141 (15)	918	<0.001
Day-care unit	325 (33)	990	7 (10)	72	318 (35)	918	<0.001
Day-care unit then to ward	33 (3)	990	7 (10)	72	26 (3)	918	0.002
Day-care unit then sent home	292 (29)	990	0 (0)	72	292 (32)	918	<0.001

Data are presented as mean values ± SD or *n* (%), as appropriate. Data derived from EHR. MACE, 30-day major adverse cardiac event.

^a^99th percentile.

### Main results

#### Performance of HEAR score calculated with computerized history taking data for major adverse cardiac event and acute coronary syndrome prediction

The HEAR score could be calculated for 666 patients of the study population (67%), using data acquired through CHT (*Figure 2*). A dropout analysis of patients with incomplete HEAR score (see Supplementary material online, *Table S3*) showed that patients with incomplete HEAR scores (*n* = 334) were older (mean age 57.1 ± 18.3 vs. 53.5 ± 16.4 years; *P* = 0.02), more likely female (52 vs. 42%; *P* = 0.03), born outside Europe (19 vs. 9%; *P* < 0.001), and retired (41 vs. 27%; *P* < 0.01). No difference was observed by type of arrival or ongoing chest pain.

**Figure 3:**
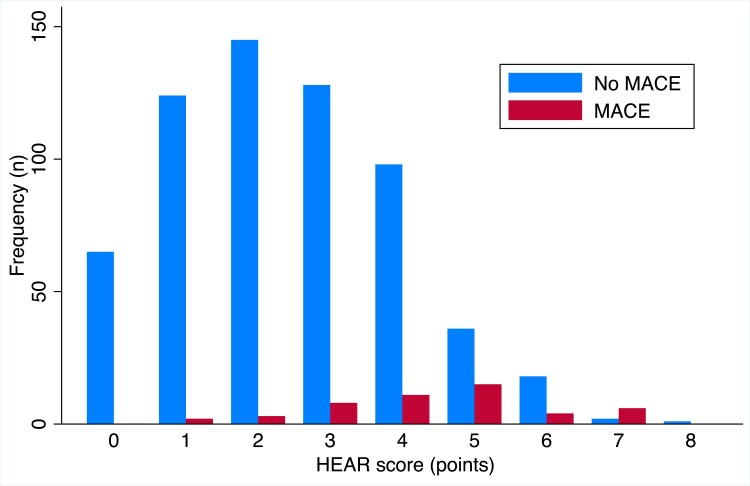
The distribution of HEAR scores in patients based on the outcome of major adverse cardiac event, showing a correlation between increasing HEAR scores and the frequency of major adverse cardiac event.

**Figure 4:**
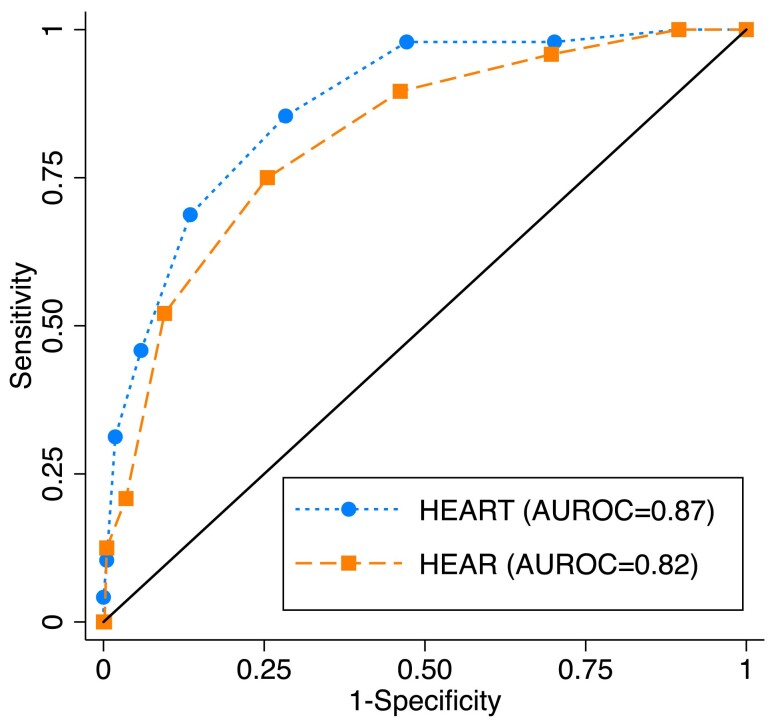
Receiver operating characteristic curves for HEART and HEAR score, for a 30-day MACE. Area under the receiver operating characteristic curve levels for both HEART and HEAR score are generally considered as excellent;^40^ however, the area under the receiver operating characteristic curve for HEART score was slightly higher (*P* < 0.01). AUROC, area under the receiver operating characteristic curve.

The distribution of HEAR score, categorized by a MACE outcome, is presented in *Figure 3*. Cross-tabulation results and the diagnostic performance of the HEAR score provided similar results for MACE and ACS, as presented in *Table 4* and in Supplementary material online, *Table S4*. A HEAR cut-off value <3 yielded an NPV comparable with <2, suggesting non-inferiority. The HEART score with cut-off value <4 had similar NPV, but slightly higher area under the receiver operating characteristic curve compared with the HEAR score (0.87 vs. 0.82; *P* < 0.01; see *Figure 4*). Major adverse cardiac event occurred in two patients with HEAR <2 and in five patients with HEAR <3, all of whom had normal ECG findings (see Supplementary material online, *Table S5* for patient characteristics). There were no adverse events reported during the CHT interviews.

**Table 4:** Performance of the HEART and HEAR scores depending on cut-off values, populated with data derived from computerized history taking for a major adverse cardiac event within 30 days

	True pos	False pos	False neg	True neg	Sensitivity	Specificity	PPV	NPV
HEART <4 *n* = 648	41	170	7	430	0.85 (0.72–0.94)	0.72 (0.68–0.75)	0.19 (0.14–0.25)	0.98 (0.97–0.99)
HEAR <3 *n* = 666	44	283	5	334	0.90 (0.78–0.97)	0.54 (0.50–0.58)	0.14 (0.10–0.18)	0.99 (0.97–1.00)
HEAR <2 *n* = 666	47	428	2	189	0.96 (0.86–1.00)	0.31 (0.27–0.34)	0.10 (0.07–0.13)	0.99 (0.96–1.00)

Data are presented as *n* or probability with 95% CI.

Pos, positive; neg, positive; PPV, positive predictive value; NPV, negative predictive value.

#### Key components in the HEAR score for major adverse cardiac event prediction

In univariate logistic regression analyses, all variables included in the HEAR score were associated with 30-day MACE. Age demonstrated the strongest correlation, followed by variables based on risk factors and history (*Table 5*). In the multivariable analysis, all variables remained associated with MACE, with age maintaining the strongest correlation. However, in this analysis, the history variable had a stronger correlation than both the ECG and risk factor variables (*Table 5*). Spearman correlation coefficients for the HEAR score components showed a moderate correlation between age and risk factors (*r* = 0.40), with all other correlations being negligible, indicating that the components were largely independent (see Supplementary material online, *Table S6*).

**Table 5:** Logistic regression analysis in patients with complete HEAR score

	Univariate analysis		Multivariable analysis	
	Odds ratio (95% CI)	*P*-value	Odds ratio (95% CI)	*P*-value
History	2.74 (1.82–4.13)	<0.001	2.38 (1.52–3.71)	<0.001
ECG	2.21 (1.51–3.24)	<0.001	1.84 (1.20–2.83)	0.005
Age	3.43 (2.13–5.52)	<0.001	2.75 (1.62–4.66)	<0.001
Risk factors	3.10 (1.96–4.90)	<0.001	1.85 (1.14–3.00)	0.012

*n* = 666.

## Discussion

This study demonstrates that using CHT to calculate HEAR score allows safe and early rule-out of 30-day MACE and ACS in two-thirds of patients presenting to the ED due to chest pain. Generating HEAR score with CHT, which is novel to this study, provides performance similar to HEAR or HEART score acquired from physician-acquired data, however with lower specificity.^7,41^ These findings suggest that the use of CHT and HEAR score could facilitate ED triage and reduce the time to identify very-low-risk patients. Such strategy could lead to fewer unnecessary troponin tests and reduce crowding in the ED, thereby prioritizing resources for patients with high-risk conditions.

Computerized history taking was accepted by most patients with chest pain in the ED, aligning with previous findings.^22,42^ Computerized history taking allows patients to provide their medical histories at their own pace during waiting times, potentially alleviating the stress and anxiety associated with recalling all details under the stressful ED conditions. Also, CHT promotes patient engagement by involving them in their own management, which may empower them and improve their overall healthcare experience.^43^ Furthermore, the systematic data collection of CHT enables consistent automated calculation of risk scores, addressing challenges related to variability in HEART score calculations between physicians,^11,12^ and the underuse of risk scores.^44^ In summary, CHT was well-accepted, even in the acute setting, enhanced the history taking process, and could potentially contribute to a more time-efficient and consistent ED triage process.

Our results indicate that a higher HEAR score cut-off (<3) may be as effective as the lower cut-off (<2) for safe rule-out of MACE, as used in most previous studies.^15^ In a study by Smith *et al.*,^45^ patients with HEAR score of <2, categorized as very low risk and excluded from troponin testing, had a low rate of missed MACE (0.9%). In a systematic review of 33 843 patients, comparing the performance of HEAR score cut-off values,^41^ a HEAR score <2 provided a pooled NPV of 1.00 (95% CI 99.7–100.0%) for MACE, consistent with our findings of an NPV of 0.99 (95% CI 0.96–1.00). Although HEAR score <3 maintained similar pooled NPV, the authors concluded, somewhat surprisingly, that HEAR score cut-off values other than <2 were not considered safe. Our analysis, also maintaining NPV with the higher cut-off (<3), suggests that this approach merits further investigation. As expected, we found that the specificity increased as the HEAR cut-off was raised from <2 to <3, with the proportion of false positives decreasing by 34% (from 428 to 283). This reduction would allow for refraining further investigations for MACE in a significant portion of patients. However, the number of false negatives, i.e. cases where a MACE outcome was missed, increased from two to five. Using CHT to calculate the HEART score would have further reduced false positives (*n* = 170) but increased false negatives (*n* = 7). Consequently, a higher NPV implies a trade-off with lower specificity. This warrants further evaluation and validation of HEAR cut-off values in diverse clinical settings, where the prevalence of MACE may differ, to ensure patient safety.

Compared with the traditional HEART score (including troponin), HEAR score (omitting troponin testing) demonstrated reduced sensitivity and increased false positives. Considering the central role of troponin in diagnosing MACE, further diagnostic evaluation, including troponin testing, is recommended for individuals with a positive HEAR score. A sequential strategy, referred to as the two-step HEART strategy, allows discharge for patients with a negative HEAR score while directing those with a positive score to further ED triage, including troponin testing. This strategy could potentially avoid troponin testing in one-fifth of patients.^46^ However, the risk of false negatives may lead to false reassurance and requires that physicians exercise caution when considering patient discharge based solely on a negative HEAR score. Given that the evidence supporting the HEAR score is limited to a few studies, caution is needed to avoid downplaying the risks of omitting biomarkers that are foundational in chest pain management. The HEART score with point-of-care troponin has shown promising results in small studies,^47,48^ however not using CHT data. Further investigation into this approach could potentially further reduce waiting times for patients with a positive HEAR score. Ultimately, any strategy must carefully balance the benefits of reducing testing and shortening ED stays against the critical need to avoid false negatives, i.e. missed diagnoses of MACE. It is important to consider the risk of false negatives in patients with high-risk characteristics, even with a low HEAR score. Finally, it is important to emphasize that CHT-derived risk scores serve as a decision-support tool rather than a directive that the physician can blindly trust. The ultimate responsibility lies with the physician, who must integrate their own expertize and judgement into the decision-making process.

Age and medical history were the most significant components of the HEAR score for predicting MACE. The significant role of medical history, also highlighted in previous studies,^49,50^ is noteworthy for two reasons: it underscores the necessity of including medical history in chest pain risk stratification tools, and it demonstrates that this critical information can be collected in an automated manner. The risk factor and ECG variables also contributed significantly, though to a lesser extent. Our results revealed no strong correlations among the variables. This indicates that each variable contributes uniquely to the risk assessment without overlapping. Overall, this study further solidifies the components of the HEAR score’s role in predicting MACE.

Smoking is a well-established risk factor for coronary artery disease.^51^ In our study, however, current smoking was not associated with an increased risk of MACE. Similar observations have been reported in previous research assessing MACE risk factors in acute settings.^2^ These findings, however, contrast with other studies that have identified smoking as a risk factor, even in acute chest pain patients.^52,53^ One possible explanation for our results is that current smokers in our study population were younger, which may have influenced the outcomes. Additionally, we observed that patients reporting ongoing chest pain during the CHT interview were less likely to experience MACE. Similarly, the group reporting ongoing chest pain was younger, but the association with non-MACE could also be due to selection bias, as those with ongoing chest pain are more likely to have ECG changes indicative of ACS. These findings on associated risk factors warrant further studies.

The main strengths of this study using self-reported CHT to collect medical histories include the large prospective cohort of ED chest pain patients, well-established and defined outcome criteria, use of already established risk stratification tools, and a generic design that allows for extrapolation to other chief complaints.

However, several limitations warrant discussion. This was a single-centre study, which introduces the potential for selection bias and may limit the generalizability of our findings. Variations in healthcare systems, ED chest pain management protocols, patient demographics, and levels of digital literacy across regions could impact the effectiveness and acceptance of CHT. However, since the CHT is unbiased and not open to the clinicians’ judgement, it may also result in a more reliable medical history.

A complete HEAR score was calculated for two-thirds of patients using CHT, while one-third did not progress far enough in the interview to provide sufficient data. The dropout analysis revealed differences in age, gender, birth region, and occupational status, extending our previous findings,^21,28^ which may introduce bias and affect the reliability of our result. Thus, further validation studies are required to confirm our findings. Notably, we reported previously that HEART score could be calculated in only one-third of patients using physician-reported EHR data,^28^ suggesting that CHT-derived risk scores may hold greater promise for automated calculations and clinical application.

The exclusion of patients unable to perform CHT, those with limited proficiency in Swedish, and critically ill patients further restricts the applicability of our results to a broader population. The rationale for excluding certain patient groups was to avoid unreliable results and to address ethical concerns (e.g. in cases of cognitive impairment) or practical issues (e.g. agitation or impaired vision). In future implementations of CHT, it is essential to identify these patient groups and provide standard history taking procedures. In addition, development of simplified and more user-friendly CHT software, also optimized for use in these patient groups, may improve accessibility.

Further, patients with limited proficiency in Swedish were excluded from this study. However, CHT can be adapted to any language, potentially addressing this limitation. Computerized history taking could promote equitable access to healthcare for individuals with limited proficiency in the local language.^54^ It may also be argued that excluding patients with more acute presentation and severe symptoms may have introduced a selection bias to our results. However, patients with compelling signs of ACS should be prioritized for immediate evaluation without determining the HEAR score, as this score is specifically designed for use in low- to intermediate-risk patients. Finally, while this study carries a potential risk of incorporation and verification bias, we believe this risk is small as patients were managed according to standard care protocols without influence from the CHT-derived HEAR or HEART scores.

Future research should address similar considerations for the CHT and HEAR score systems as those highlighted by Giannitsis *et al*.^55^ for high-sensitivity point-of-care testing. Before routine care clinical implementation, prospective multicentre validation across diverse medical environments is needed to explore long-term implications, including patient management, patient outcomes, cost-effectiveness, patient satisfaction, and physician acceptance. Additionally, the impact of CHT on risk prediction for other serious causes of chest pain, e.g. aortic dissection or pulmonary embolism, requires further investigation. Further, a substantial portion of ED patients are discharged without a clear explanation for their symptoms. Developing strategies to improve the diagnosis of benign causes could alleviate unnecessary pain and anxiety. Future studies should also evaluate the two-step HEART strategy using CHT data. As this method does not initially require troponin testing, it could be employed in a pre-hospital setting to rule-out MACE in a selected group of patients and allow very-low-risk patients to refrain ED evaluation. However, the balancing act between troponin use and early discharge must be acknowledged to avoid false negatives. This study suggests further exploration of the HEAR score <3, which should be validated and compared with the <2 cut-off. Further development of the software focusing only on critical questions could shorten the interview duration and makes the tool more useful. Improving the usability of CHT software is necessary to provide sufficient data for risk determination for more than two-thirds of patients and for patients excluded in this study. Additional technical development could enhance CHT’s accessibility and usability, with features like QR code scanning for interview access and user interface improvements.

## Conclusions

The use of CHT to calculate a HEAR score in patients with chest pain in the ED can effectively identify those at very low risk, facilitating safe rule-out of 30-day MACE and ACS. Within the HEAR score, age and chest pain characteristics emerged as the most significant predictors. These findings may reduce unnecessary clinical investigations and hospital admissions for the substantial number of very-low-risk chest pain patients in the ED and influence pre-hospital strategies.

## Lead author biography


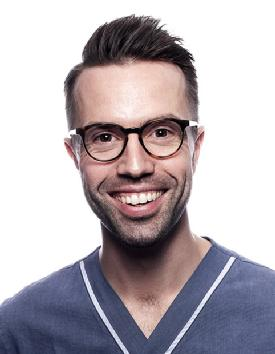


Dr Helge Brandberg, MD, from Linköping University (2010), a cardiologist at the Department of Cardiology, Danderyd University Hospital, Stockholm, where he serves as the Medical Informatics Officer. He is a PhD Candidate at Karolinska Institutet, Division of Cardiovascular Medicine, Department of Clinical Sciences, Danderyd Hospital. His interdisciplinary clinical research within cardiology, health informatics, and medical management reflects his passion for integrating innovative technology into clinical practice.

## Supplementary Material

ztae087_Supplementary_Data

## Data Availability

The data that support the findings of this study are available from the corresponding author, H.B., upon reasonable request.
