# Supplementary material for: Performance of computerized self-reported medical history taking and HEAR score for safe early rule-out of cardiac events in acute chest pain patients: the CLEOS-CPDS prospective cohort study
Source: Eur Heart J Digit Health. 2024 Nov 12;6(1):104–14. doi: 10.1093/ehjdh/ztae087 (PMC11750193; doi:10.1093/ehjdh/ztae087)
Supplement: ztae087_Supplementary_Data [file ztae087_supplementary_data.doc]

Performance of Computerised Self-Reported Medical History-Taking and HEAR Score for Safe Early Rule-Out of Cardiac Events in Acute Chest Pain Patients: The CLEOS-CPDS Prospective Cohort Study

**Supplementary material**

**Supplementary Tables**

[**Supplementary Table 1.** Demography and background of all included patients divided into groups depending on ACS outcome or not. 2](#__RefHeading___Toc169204604)

[**Supplementary Table 2.** Vital signs, electrocardiogram, circulating biomarkers and disposition of all included patients divided into groups depending on ACS outcome or not. 3](#__RefHeading___Toc169204605)

[**Supplementary Table 3.** Dropout analysis between groups with complete or incomplete HEAR score. 4](#__RefHeading___Toc169204606)

[**Supplementary Table 4.** Performance of the HEART and HEAR scores depending on cut-offs, populated with data derived from computerised history taking for an acute coronary syndrome within 30 days. 5](#__RefHeading___Toc169204607)

[**Supplementary Table 5.** Characteristics of false negative patients. 6](#__RefHeading___Toc169204608)

[**Supplementary Table 6.** Spearman correlation for the components in the HEAR score (n=666). 7](#__RefHeading___Toc169204609)

**Supplementary Figure**

[**Supplementary Figure 1.** Missing data per HEART score variable and patterns of missing data, annotated with the patient count for each variable. Blue color indicates available data and red color indicates missing data. 8](#__RefHeading___Toc167888764)

**Supplementary Table 1.** Demography and background of all included patients divided into groups depending on ACS outcome or not.

|  | **All** |  | **ACS** |  | **Non-ACS** |  | **P-value** |
| --- | --- | --- | --- | --- | --- | --- | --- |
| **Characteristic** | Value | n | Value | n | Value | n |  |
| Age, years | 54.7 ± 17.2 | 1000 | 68.0 ± 10.9 | 64 | 53.9 ± 17.1 | 930 | <.001 |
| Sex (females) | 456 (46) | 1000 | 18 (28) | 64 | 436 (46) | 930 | .004 |
| Body mass index, kg/m2 | 26.4 ± 4.7 | 1000 | 26.2 ± 3.9 | 64 | 26.4 ± 4.7 | 928 | .813 |
| Diabetes mellitus type 1 or 2 | 62 (8) | 788 | 5 (11) | 44 | 57 (8) | 740 | .382 |
| Ongoing lipid lowering medication | 128 (20) | 640 | 18 (46) | 39 | 110 (18) | 597 | <.001 |
| Hypertension | 316 (41) | 765 | 31 (67) | 46 | 285 (40) | 715 | <.001 |
| Family history of coronary artery disease | 200 (26) | 774 | 17 (41) | 41 | 183 (25) | 729 | .020 |
| Known coronary artery disease | 137 (16) | 851 | 27 (55) | 49 | 110 (14) | 798 | <.001 |
| History of angina pectoris | 88 (10) | 850 | 20 (41) | 49 | 68 (9) | 797 | <.001 |
| History of myocardial infarction | 82 (10) | 850 | 18 (37) | 49 | 64 (8) | 797 | <.001 |
| History of percutaneous coronary  intervention | 76 (9) | 833 | 13 (27) | 48 | 63 (8) | 781 | <.001 |
| History of coronary artery bypass graft | 18 (2) | 833 | 9 (19) | 48 | 9 (1) | 781 | <.001 |
| Current smoker | 90 (11) | 790 | 5 (11) | 46 | 84 (11) | 740 | .920 |
| Region of birth |  |  |  |  |  |  |  |
| Nordic countries | 829 (83) | 1000 | 55 (86) | 64 | 769 (83) | 930 | .504 |
| Europe (outside the Nordic countries) | 46 (5) | 1000 | 1 (2) | 64 | 45 (5) | 930 | .228 |
| Outside Europe | 125 (13) | 1000 | 8 (13) | 64 | 116 (12) | 930 | .995 |
| Occupational status |  |  |  |  |  |  |  |
| Active worker (employed, student) | 616 (62) | 1000 | 23 (36) | 64 | 589 (63) | 930 | <.001 |
| Not at work (unemployed, on sick  leave) | 69 (7) | 1000 | 1 (2) | 64 | 67 (7) | 930 | .084 |
| Retired | 315 (32) | 1000 | 40 (63) | 64 | 274 (29) | 930 | <.001 |
| Arrived at ED by ambulance | 189 (21) | 917 | 13 (25) | 51 | 176 (20) | 862 | .385 |
| Ongoing chest pain during CHT | 544 (61) | 892 | 16 (33) | 48 | 524 (62) | 840 | <.001 |

Data are presented as mean values ± SD or n (%), as appropriate. Self-reported medical history data derived from CHT. ACS: 30-day acute coronary syndrome. CHT: computerised history-taking. ED: emergency department.

**Supplementary Table 2. Vital signs, electrocardiogram, circulating biomarkers and disposition of all included patients divided into groups depending on ACS outcome or not.**

|  | **All** |  | **ACS** |  | **Non-ACS** |  | **P-value** |
| --- | --- | --- | --- | --- | --- | --- | --- |
| **Characteristic** | Value | n | Value | n | Value | n |  |
| Vital parameters at triage |  |  |  |  |  |  |  |
| Systolic blood pressure, mmHg | 143 ± 22 | 995 | 148 ± 22 | 64 | 143 ± 22 | 926 | .081 |
| Diastolic blood pressure, mmHg | 83 ± 13 | 993 | 81 ± 15 | 63 | 83 ± 13 | 910 | .178 |
| Heart rate | 77 ± 16 | 984 | 74 ± 16 | 64 | 77 ± 16 | 913 | .193 |
| Respiration rate | 16 ± 3 | 984 | 16 ± 2 | 64 | 16 ± 3 | 915 | .861 |
| Body temperature (°C) | 36.8 ± 0.4 | 947 | 36.6 ± 0.4 | 63 | 36.8 ± 0.4 | 879 | .001 |
| Electrocardiogram |  |  |  |  |  |  |  |
| New signs diagnostic for ischemia | 68 (7) | 994 | 14 (22) | 63 | 53 (6) | 926 | <.001 |
| New nonspecific ST-T changes | 131 (13) | 994 | 16 (25) | 63 | 115 (12) | 926 | .003 |
| Normal or known ST-T alterations | 795 (80) | 994 | 33 (52) | 63 | 758 (82) | 926 | <.001 |
| High sensitive troponin T values |  |  |  |  |  |  |  |
| >3 x normal limit (> 42 ng/L) | 47 (5) | 970 | 28 (44) | 63 | 19 (2) | 901 | <.001 |
| 1-3 x normal limit (15-42 ng/L | 112 (12) | 970 | 16 (25) | 63 | 96 (11) | 901 | <.001 |
| Normal limit* (≤14 ng/L) | 811 (84) | 970 | 19 (30) | 63 | 786 (86) | 910 | <.001 |
| 5-14 ng/L | 397 (41) | 970 | 11 (17) | 63 | 368 (41) | 901 | <.001 |
| 1 h troponin T elevated (>2 ng/L) | 21 (2) | 970 | 2 (3) | 63 | 19 (2) | 901 | .575 |
| <5 ng/L | 414 (43) | 970 | 4 (6) | 63 | 407 (45) | 901 | <.001 |
| Admitted to the ward or day-care unit | 528 (53) | 990 | 62 (97) | 64 | 466 (50) | 926 | <.001 |
| Ward (not via day-care unit) | 203 (21) | 990 | 57 (89) | 64 | 146 (16) | 926 | <.001 |
| Day-care-unit | 325 (33) | 990 | 5 (8) | 64 | 320 (35) | 926 | <.001 |
| Day-care unit then to ward | 33 (3) | 990 | 5 (8) | 64 | 28 (3) | 926 | .039 |
| Day-care unit then sent home | 292 (29) | 990 | 0 (0) | 64 | 292 (32) | 926 | <.001 |

Data are presented as mean values ± SD or n (%), as appropriate. Data derived from EHR. MACE: 30-day major adverse cardiac event. ACS: acute coronary syndrome. *99th percentile. For definitions of interpretation of electrocardiograms see supplementary material (Supplementary material, Table 1)

**Supplementary Table 3. Dropout analysis between groups with complete or incomplete HEAR score.**

|  | **Complete** |  | **Incomplete** |  | **P-value** |
| --- | --- | --- | --- | --- | --- |
| **Characteristic** | Value | n | Value | n |  |
| Age, years | 53.5 ± 16.4 | 666 | 57.1 ± 18.3 | 334 | .002 |
| Sex (females) | 282 (42) | 666 | 174 (52) | 334 | .003 |
| Body mass index, kg/m2 | 26.7 ± 4.7 | 666 | 25.7 ± 4.6 | 334 | .002 |
| Region of birth |  |  |  |  |  |
| Nordic countries | 573 (86) | 666 | 256 (77) | 334 | <.001 |
| Europe (outside the Nordic countries) | 30 (5) | 666 | 16 (5) | 334 | .839 |
| Outside Europe | 63 (9) | 666 | 62 (19) | 334 | <.001 |
| Occupational status |  |  |  |  |  |
| Active worker (employed, student) | 441 (66) | 666 | 175 (52) | 334 | <.001 |
| Not at work (unemployed, on sick  leave) | 46 (7) | 666 | 23 (7) | 334 | .990 |
| Retired | 179 (27) | 666 | 136 (41) | 334 | <.001 |
| Arrived at ED by ambulance | 127 (19) | 665 | 62 (25) | 252 | .066 |
| Ongoing chest pain during CHT | 410 (62) | 665 | 134 (59) | 227 | .484 |
| Admitted to the ward or day-care unit | 361 (55) | 658 | 167 (50) | 332 | .174 |
| Ward (not via day-care unit) | 126 (19) | 658 | 77 (23) | 332 | .137 |
| Day-care-unit | 235 (36) | 658 | 90 (27) | 332 | .006 |
| Day-care unit then to ward | 215 (33) | 658 | 77 (23) | 332 | .002 |
| Day-care unit then sent home | 20 (3) | 658 | 13 (4) | 332 | .468 |

Data for the study population (n=1000) are presented as mean values ± SD or n (%), as appropriate. Self-reported demographic data derived from CHT. Data on disposition derived from EHR. Missing data for ambulance arrival (n=83), ongoing chest pain (n=108), and disposition (n=10). CHT: computerised history-taking. ED: emergency department.

**Supplementary Table 4. Performance of the HEART and HEAR scores depending on cut-offs, populated with data derived from computerised history taking for an acute coronary syndrome within 30 days.**

|  | **Truepos** | **False pos** | **False neg** | **True neg** | **Sensitivity** | **Specificity** | **PPV** | **NPV** |
| --- | --- | --- | --- | --- | --- | --- | --- | --- |
| **HEART <4**  n=648 | 35 | 176 | 6 | 431 | 0.85  (0.71–0.94) | 0.71  (0.67–0.75) | 0.17  (0.12–0.22) | 0.99  (0.97–1.00) |
| **HEAR <3**  n=666 | 38 | 289 | 4 | 335 | 0.91  (0.77–0.97) | 0.54  (0.50–0.58) | 0.12  (0.08–0.16) | 0.99  (0.97–1.00) |
| **HEAR <2**  n=666 | 40 | 435 | 2 | 189 | 0.95  (0.84–0.99) | 0.30  (0.27–0.34) | 0.08  (0.06–0.11) | 0.99  (0.96–1.00) |

pos=positive, neg=positive, PPV=positive predictive value, NPV=negative predictive value.

Data are presented as n or probability with 95% confidence interval.

**Supplementary Table 5. Characteristics of false negative patients.**

| **Patient** | **Age (years)** | **Sex** | **HEAR score** | **ECG findings** | **Type of MACE** |
| --- | --- | --- | --- | --- | --- |
| 1 | 58 | Male | 1 | Normal | Acute myocardial infarction |
| 2 | 63 | Female | 1 | Normal | Acute myocardial infarction |
| 3 | 48 | Female | 2 | Normal | Revascularization within 30 days (no ACS diagnosis) |
| 4 | 62 | Male | 2 | Normal | Acute myocardial infarction |
| 5 | 67 | Male | 2 | Normal | Acute myocardial infarction |

ECG=electrocardiogram, MACE=major adverse cardiac event, ACS=acute coronary syndrome

**Supplementary Table 6. Spearman correlation for the components in the HEAR score (n=666).**

|  | **History** | **ECG** | **Age** | **Risk factors** |
| --- | --- | --- | --- | --- |
| **History** | 1.00 | N/A | N/A | N/A |
| **ECG** | 0.08 | 1.00 | N/A | N/A |
| **Age** | 0.04 | 0.13 | 1.00 | N/A |
| **Risk factors** | 0.13 | 0.15 | 0.40 | 1.00 |

P=.25 for History and Age, and P<.05 for all other comparisons.

**Supplementary Figure 1.** Missing data per HEART score variable and patterns of missing data, annotated with the patient count for each variable. Blue colour indicates available data and red colour indicates missing data.
